# Report of the Cases Treated by Kousso, in King’s College Hospital

**Published:** 1850-11

**Authors:** 


Tape-Worm.—[As the new Anthelmintic Kousso is now being tested extensively in Europe, our readers will be gratified with the following clinical lecture on the subject. Our correspondent, who, in view of the enormous price of this remedy, wishes attention directed to the cheap and efficacious substitute found in Sp. Terebinth, will perceive that the new agent succeeds after the turpentine has failed. Whether a perseverance in the latter remedy might not have been completely successful, may be highly problematical.]—*New-York Medical Gazette.*

*Report of the Cases Treated by Kousso, in King's College Hospital.* By Dr. Budd.

Gentlemen,—We have lately been employing in the hospital, a new remedy for tape-worm, the "Kousso." Our stock of it is now exhausted, and it is right that you should know the amount of evidence that our experience has furnished in its favor. "Kousso," otherwise called "Brayera Anthelmintica," from Dr. Brayer, who first made its virtues known in Europe, is a tree, growing in Abyssinia, which has been classed among the *Rosaceæ,* and which is said to attain the height of an oak, and to bear large bunches of very small flowers, varying from a pale green to a rose color. The flowers, which are the medicinal part of the plant, are used by the natives of Abyssinia as a remedy for tape-worm, which is very common among them. The medicine comes to us a brownish powder, looking very like jalap, smelling very like scammony, and slightly bitter and somewhat nauseous to the taste. It can, I believe, be procured at present only from a single druggist—M. Boggio, pharmacien, 13, Rue Neuve des Petits Champs, Paris—who charges the enormous price of forty francs, that is, thirty-five shillings, for each dose of it, which weighs four drachms and a half, and is contained in a well-stopped bottle.*

Some time ago I obtained four doses of it through the kindness of my friend, Dr. de Mussy. I let Dr. Marshall Hall have one of these, and gave the other three to patients who came to the hospital with tape-worm. The medicine having proved successful in each of these three cases, six more doses of it were got from Paris, on account of the hospital. Three of these have been used by myself; the rest by my colleague, Dr. Todd. Nine doses of the medicine have been given by us, and not one of them has failed to kill and expel the worm.

The medicine has been given in the morning, before breakfast, which is the best time for administering all remedies for tape-worm, as the small intestine which the worm inhabits, is then more empty than at other times; the worm, consequently, less likely to be sheathed and protected from the action of the drug by the other contents of the bowel.

The powder has been infused for ten minutes in three-fourths of a pint of hot water. The infusion has then been stirred, and the whole drank.

One of the patients under the care of Dr. Todd, a woman advanced in pregnancy, vomited about half of the dose; but what remained in the stomach destroyed the worm. Two of the other patients had a slight feeling of nausea for ten minutes or a quarter of an hour after taking the

* Mr. Hooper, Druggist, 7, Pall-mall East, has obtained some of the Kousso from Paris, which he sells to hospitals for 16s. and to the public for 20s. a dose.—M. Delluc, Broadway, New-York, has a supply, which he sells at $10 the dose.

medicine. Another was several times purged by it. Another, the woman to whom we first gave it, had headache after taking it, and ascribed it to a diuretic effect. The rest felt no uneasy sensations whatever from the medicine.

In the cases that fell under my own care, I ordered the patients to live sparingly, and to take a seidlitz powder, or a dose of castor oil, the day before taking the Kousso, for the sake of emptying the bowels, and so leaving the worm more exposed. This is a precaution which it is well to take before administering any of the remedies for tape-worm. They all act directly on the worm, and to take effect must, of course, be brought in contact with it.

I also ordered the patients to take a seidlitz powder, or a dose of castor oil, after the Kousso, to carry the medicine down to the worm, and to expel the dead or enfeebled worm from the bowels. This expedient also is equally applicable to other remedies for tape-worm, and especially to turpentine, which, if it remains long in the stomach, or passes slowly through the bowels, gets absorbed and irritates the kidneys and bladder, producing strangury and bloody urine, and may not reach the worm in quantity sufficient to kill it.

Tape-worms are very tenacious of life. They are seldom voided entire without the aid of medicines that act especially upon them. Single joints often come away, and pieces two or three feet long are often then voided, but it very seldom happens, unless after medicine, that a portion comprising the head is thus passed. This portion remains and grows again, so that a person is often plagued with the parasite for years. One of our patients, Sarah Wheeler, had been so plagued for sixteen years, during which she told us that she had seldom gone a week without passing joints of the worm. She had taken turpentine, and the bark of the pomegranate root ; and some years ago, when she was at Fort Beaufort, at the Cape of Good Hope, she took the root of a plant used by the natives as a remedy for tape-worm, which, she says, is called there "Cacay." She describes this root as being round, and when scraped, white like a turnip, and sweet to the taste. This medicine had very little effect. The turpentine and the pomegranate brought away large pieces of the worm, but the head and the portion near it remained, and the worm grew again. The worm was expelled after the kousso in different portions, on the 11th and 12th of April, and she has since had none of the symptoms which she attributed to it. She is still (May 31st) in the hospital, for prolapsus of the uterus, which she has long had, but she feels convinced that the worm is entirely destroyed.

Another case that shows more strikingly still the tenacity of life of the tape-worm, is that of Samuel Payne. He first passed joints of the worm seven years ago. In September last, he was brought into the hospital with cholera, and on the day of his admission, passed a portion of the worm two yards in length. He remained in the hospital three weeks, on account of the cholera. Some time after this he again passed joints of the worm, and to get rid of it, came to the hospital as an out-patient. Turpentine and castor oil were given him, and brought away a long piece of the worm. The ailments which he attributed to the worm were much relieved for a time. They then became again more severe ; joints of the worm were again passed, and in January last he came for the second time to the hospital to be rid of the worm. Turpentine and castor oil were given him, as before a long piece of the worm was again expelled, but the creature was not destroyed; so that here the worm had existed seven years, had kept its place during the terrible commotion and flooding of the intestines in cholera, and had escaped destruction by two doses of turpentine. Payne took the kousso on the 3d of May, and the next morning voided the worm, which was ten yards long.

In all the cases in which the kousso had been given in the hospital, the head of the worm, or the taper portion near the head, has been found, so that there is reason to suppose that the creature has been entirely destroyed. The action of the medicine is very speedy. In one of the cases, in which the bowels were slow to move, the worm was expelled, in different portions, in the two days succeeding that in which the kousso was taken; in all the other cases it was expelled the same day, and in several of them after the lapse of only three or four hours.

The result of our experience then, is, that the kousso is a very effectual remedy for the tape-worm, and that in its action it is both speedy and safe.

I have had little experience of the male fern. But the kousso is certainly much more efficacious than the pomegranate ; and much more efficacious, as well as less disagreeable, and safer, than turpentine. There is a considerable demand for it at the hospital, which, on account of the cost, we cannot satisfy. I trust, however, now that its efficacy is known in this country, which has commercial relations with every part of the world, that a fresh supply will soon be brought here, and that we shall have it at a reasonable rate.

From one of our patients two worms, one very large, and the other much smaller, were expelled by the kousso. It sometimes happens that as many as three or four of these worms are found together; but such an event is very uncommon. The tape-worm requires no mate, and generally leads a solitary life; and, hence, has been called by the French *ver solitaire*, the solitary worm.

In the cases which fell under my own care at the hospital, notes were taken of the ailments which the patients attributed to the worm. Most of these persons had some unpleasant sensations referred to the stomach and bowels, such as a feeling of sickness or nausea, especially on first getting up in the morning ; pain, or a sense of sinking at the epigastrium ; flatulence ; a feeling at times as if the worm were moving in the bowel. It is worthy of remark, that in none of them was there diarrhœa. The appetite was very variable—in some, much impaired, in others, craving.

Two or three of the patients had a troublesome cough, and one, annoying palpitation ; which were ascribed to the worm. Some of them complained also of unpleasant sensations in the head ; giddiness or drowsiness felt during the day, and especially after meals ; and almost all complained, more or less, of weakness, and lassitude, and incapacity for work.

The most constant of the symptoms were a feeling of lassitude, a sense of nausea on first getting up in the morning, and drowsiness felt during the day. But symptoms such as these, are of very little use in diagnosis. Persons who have tape-worm generally discover the fact by passing of joints. The worm seems to grow rapidly ; and the tail-joints, as they attain their full development, drop off, and are voided with the other contents of the bowel. Probably no person suffers very long from tapeworm without voiding some of these joints. The existence of the worm cannot be safely inferred, and the remedies for it cannot be given with a fair chance of success, unless joints have been passed. It is a common belief that while joints are dropping, the worm is feeble, and that the medicines which kill it act more surely soon after joints have been passed. —*London Lancet.*

